# Influence of Hydrolysis on Non-Isothermal Crystallization of Poly(Butylene Succinate-Co-Adipate) (PBSA)

**DOI:** 10.3390/molecules30112252

**Published:** 2025-05-22

**Authors:** Anna Svarcova, Marie Dvorackova, Petr Svoboda

**Affiliations:** 1 Department of Polymer Engineering, Faculty of Technology, Tomas Bata University in Zlin, Vavreckova 5669, 76001 Zlin, Czech Republic; a_svarcova@utb.cz; 2 Department of Environmental Protection Engineering, Faculty of Technology, Tomas Bata University in Zlin, Nad Ovcirnou 3685, 76001 Zlin, Czech Republic; dvorackova@utb.cz

**Keywords:** poly(butylene succinate-co-adipate) (PBSA), hydrolysis, non-isothermal crystallization, molecular weight distribution, crystallization kinetics, crystallization morphology, spherulites, degradation, biodegradable polyesters

## Abstract

This study investigates the impact of hydrolysis on the crystallization behavior of poly(butylene succinate-co-adipate) (PBSA), a biodegradable polyester. Hydrolysis was conducted in a controlled environment using phosphate-buffered saline at 70 °C to isolate the impact of hydrolytic degradation on the polymer’s properties. The consequent changes in molecular weight characteristics were tracked using gel permeation chromatography (GPC), revealing a decrease in both weight average molecular weight (M_w_) and an increase in polydispersity index (PDI) as hydrolysis progressed. The thermal behavior of PBSA during hydrolysis was thoroughly investigated using differential scanning calorimetry (DSC), which demonstrated significant changes in melting temperature (T_m_), glass transition temperature (T_g_), and crystallinity (X). These changes in T_m_ and T_g_ suggest a change in copolymer composition, likely due to the greater susceptibility of the adipic acid unit to hydrolysis compared to the succinic acid unit. Furthermore, polarized optical microscopy (POM) was employed to observe the morphological evolution of PBSA, showing a transition from spherulitic structures in the early stages of hydrolysis to dendritic structures with prolonged hydrolysis time. The decrease in nucleation activity led to a reduction in the number of spherulites, which in turn allowed the remaining spherulites to grow larger.

## 1. Introduction

The escalating environmental concerns associated with persistent plastic waste have intensified the pursuit of biodegradable polymers as sustainable alternatives [1,2,3]. Among these, poly(butylene succinate-co-adipate) (PBSA), an aliphatic polyester synthesized from succinic acid and 1,4-butanediol, has attracted significant attention due to its favorable mechanical properties, flexibility, and biodegradability [4,5]. PBSA’s applications span various sectors, including agriculture, packaging, and biomedical engineering, where controlled degradation is often a required [6,7,8].

Recent research has increasingly focused on enhancing PBSA properties and biodegradability through the development of composites [7,9,10,11] and polymer blends [12,13,14]. Altieri et al. [15] demonstrated that incorporating collagen hydrolysates derived from the tanning industry can improve both the mechanical performance and biodegradation rate of PBSA in composting environments. Similarly, Strangis et al. [16] reported that the addition of wheat bran, a low-cost and hygroscopic by-product from the agri-food sector, improved the marine biodegradability of PBSA composites. This effect was attributed to the swelling behavior of bran, which promoted matrix fragmentation and increased the polymer–seawater interfacial area, thereby accelerating degradation. In agricultural applications, Cappello et al. [17] explored the use of biochar, a carbonaceous filler obtained through pyrolysis of woody biomass waste, as a sustainable alternative to conventional carbon black. Their findings revealed that biochar-filled PBSA composites exhibit favorable mechanical properties and environmental performance, with potential fertilizing benefits for agricultural applications. Thummarungsan et al. [18] developed magneto-responsive PBSA materials by embedding Fe_3_O_4_ nanoparticles, showing promise for soft robotics and biomedical actuation. In a related study, the same authors designed electroactive PBSA-based materials by incorporating conductive poly(o-phenylenediamine) and a plasticizer, paving the way for flexible biodegradable actuators in artificial muscles, sensors, and wearable electronics [19]. Studies have also explored PBSA degradation behavior in diverse environments, such as marine settings, to assess its suitability for broader ecological applications [20]. However, these investigations often emphasize enzymatic degradation pathways or the behavior of PBSA in composite forms, leaving a gap in understanding the hydrolytic degradation mechanisms of neat PBSA under varying thermal conditions.

Hydrolytic degradation, a process wherein water molecules cleave ester bonds in the polymer backbone, is a critical pathway for the breakdown of aliphatic polyesters like PBSA [21]. This process is influenced by multiple factors, including temperature, pH, environmental moisture, and the presence of catalysts or enzymes [22,23,24]. Among these, crystallinity plays a central role in determining the polymer’s susceptibility to degradation. Crystalline regions are densely packed and restrict water penetration, thereby slowing hydrolytic and microbial activity. In contrast, amorphous regions are more disordered and flexible, allowing easier access for water and enzymes, and thus degrade more readily. Additionally, the melting temperature (T_m_) of the polymer is a relevant indicator of its thermal stability and degradation potential. Polymers with higher T_m_ are generally more resistant to hydrolysis [25]. The physical dimensions of the material further affect the degradation rate, as smaller or thinner structures expose more surface area to degrading agents [26]. While enzymatic degradation may be effective under specific conditions, elevated temperatures can significantly accelerate hydrolytic reactions, even in the absence of enzymatic activity [25,27,28]. For instance, La Fuente et al. [25] have demonstrated that degradation was slower in soil and liquid media than in compost, largely attributable to the lower temperatures in those environments. An increasing the temperature from 50 °C to 70 °C markedly enhances the hydrolysis rate of aliphatic polyesters, underscoring the importance of thermal conditions in degradation kinetics.

PBSA exhibits a relatively low melting point of approximately 80 °C, which may pose limitations for certain applications, but offers the benefit of reduced energy requirements during processing [29]. Despite these insights, there remains a lack of systematic studies examining the hydrolytic degradation of neat PBSA at elevated temperatures, particularly at 70 °C. Most existing research concentrates on lower temperature ranges or investigates PBSA within composite materials, thereby limiting the understanding of its intrinsic degradation behavior under thermally accelerated conditions. This knowledge gap is critical, especially for applications where PBSA is exposed to higher temperatures, necessitating a comprehensive understanding of its stability and degradation profile.

Addressing this gap, the present study investigates the hydrolytic degradation of neat PBSA at three distinct temperatures: 37 °C, 58 °C, and 70 °C, with a particular focus on the highest temperature due to the pronounced degradation trends observed. The experiments were conducted in phosphate-buffered saline (PBS) at pH 7.2 to simulate near-neutral aqueous conditions. The selection of 70 °C was not arbitrary; it aligns with typical thermophilic composting conditions, where temperatures commonly range between 50 °C and 70 °C. This makes the findings particularly relevant for evaluating PBSA’s behavior in industrial composting scenarios. Although hydrolysis of aliphatic polyesters is often more pronounced under acidic conditions, we selected a pH of 7.2 to isolate the effect of elevated temperature, which has been shown to significantly influence hydrolytic degradation kinetics. For context, soil pH values typically range from 3.5 to 7.1, meaning our selected conditions remain within the broader spectrum of environmentally relevant parameters.

This research aims to investigate the properties of PBSA by focusing on degree of hydrolysis at elevated temperature monitored by gel permeation chromatography (GPC). Differential scanning calorimetry (DSC) will be used to observe the changes in melting temperature (T_m_), glass transition temperature (T_g_), crystallinity (X) and crystallization kinetics. Furthermore, polarized optical microscopy (POM) will be employed to observe spherulitic structures development during hydrolysis. We seek to gain a deeper understanding of the degradation mechanism and how to accelerate the degradation process.

Previous studies have examined PBSA biodegradation at temperatures lower than 70 °C (e.g., 30 °C and 50 °C) [30,31]. However, our study uniquely investigates the effects of biodegradation at 70 °C.

## 2. Results and Discussion

### 2.1. Hydrolysis

#### Compost

A thermophilic composting process can achieve temperatures ranging from 49 to 77 °C within several days. With optimal management, the decomposition of organic matter can be completed in approximately four weeks. The exothermic heat generated during this process has the potential for thermal energy recovery in applications such as water heating or climate control for residential or horticultural structures. However, rigorous temperature monitoring is crucial, as sustained exposure to temperatures exceeding 65 °C can lead to the devitalization of beneficial mesophilic and thermophilic microorganisms essential for efficient decomposition [32].

The influence of temperature on the hydrolysis of poly(butylene succinate-co-adipate) (PBSA) is a key consideration in understanding its degradation behavior. Elevated temperatures significantly accelerate the rate of ester bond cleavage [33], the fundamental chemical reaction driving hydrolysis. This occurs because increased thermal energy enhances the kinetic energy of both water molecules and the polymer chains, leading to more frequent and energetic collisions that effectively overcome the activation energy barrier for the hydrolytic reaction. Consequently, higher temperatures result in a faster rate of polymer chain fragmentation and a more rapid decrease in molecular weight. Furthermore, the increased mobility of polymer chains at higher temperatures can improve the accessibility of ester bonds to water molecules. Importantly, temperature can also affect the relative hydrolysis rates of the succinate and adipate units within the PBSA copolymer, potentially leading to changes in the material’s chemical composition as the degradation process progresses.

**Figure 1:**
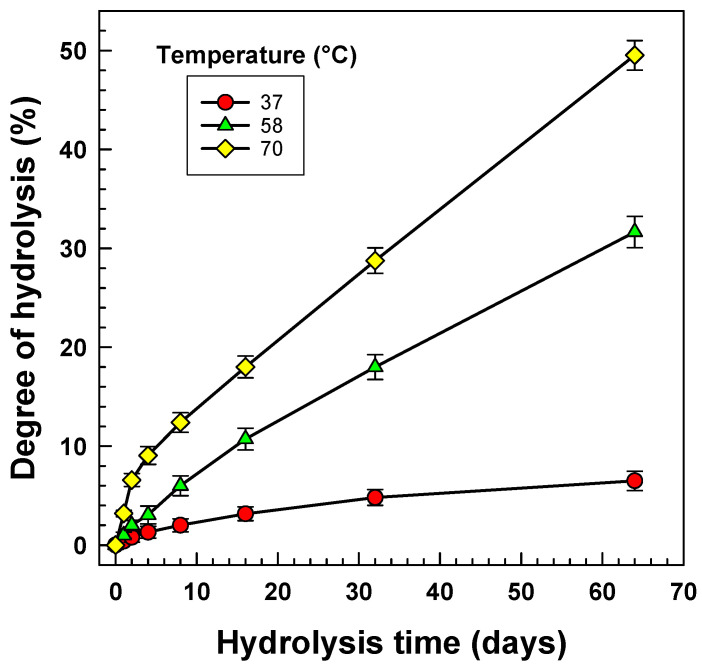
Degree of hydrolysis of PBSA during hydrolysis at various temperatures.

Figure 1 illustrates the degree of hydrolysis of poly(butylene succinate-co-adipate) (PBSA) as a function of time at temperatures of 37 °C, 58 °C, and 70 °C. While degree of hydrolysis at 37 °C is very low, at 58 °C it is much higher and 70 °C is the highest of these three temperatures. After 64 days of hydrolysis the values of degree of hydrolysis were 6.5, 31.7 and 49.5% for temperatures of 37, 58 and 70 °C, respectively. We have analyzed the slope of the lines between 16 and 64 days and the values were 0.0671, 0.4351 and 0.6556 for temperatures of 37, 58 and 70 °C, respectively. Wang et al. [34] in 2020 also observed that at higher temperatures the hydrolysis proceeds faster. Wang et al. [35] in 2022 evaluated PLA and its copolymers with glycolic acid with various copolymer composition PLGA. They observed similar trend of increasing degree of hydrolysis in water and in soil with increasing hydrolysis time, which was higher for copolymers compared to virgin PLA.

The temperature of 37 °C is frequently employed in enzymatic hydrolysis investigations, consistent with the methodology used by Tsuji et al. [36] in their study of poly(L-lactic acid) (PLLA) film hydrolysis in phosphate-buffered solution.

Similar trends in hydrolysis kinetics were observed by Bikiaris et al. [37] who investigated the biodegradability of three poly(alkylene succinate)s. Notably, Bikiaris et al. reported a normalized weight loss of 9 mg/cm^2^ for poly(butylene succinate) (PBSu) after 64 days, a value that aligns closely with our findings.

The rate of hydrolysis is primarily influenced by reaction temperature and enzyme species. For aliphatic polyesters, an increase in temperature, approaching the polymer’s melting point, accelerates enzymatic hydrolysis. Consequently, optimal hydrolysis rates are generally achieved at temperatures 10–20 °C below the polymer’s melting point.

Bikiaris et al. [37] reported that enzymatic degradation of poly(butylene succinate) by Pseudomonas species at pH 7.0 was undetectable after 20 h at 50 °C, leading to the conclusion that it was not biodegradable under those conditions. Moreover, elevated temperatures can induce chemical hydrolytic cleavage of ester bonds, independent of enzymatic activity [37].

A significant increase in degradation rate with temperature is evident. Marten et al. [38] examined the degradation of aliphatic polyesters, including adipic and succinic acid-based polymers, at 25 °C, 37 °C, and 50 °C. Their study, conducted over a 20-h period, revealed that certain polyesters, specifically those containing succinic acid, exhibited no degradation within the experimental timeframe.

The 64-day duration of our hydrolysis experiments, while exceeding the typical four-week active thermophilic composting phase focused on rapid organic matter decomposition, was specifically chosen to investigate the longer-term chemical degradation of the PBSA material under controlled hydrolytic conditions. This extended timeframe enables observation of the progressive ester bond breakdown and quantification of the degree of hydrolysis over a substantial period, providing crucial insights into the polymer’s inherent susceptibility to hydrolytic degradation relevant to its environmental fate in various applications involving prolonged exposure to moisture and fluctuating temperatures, a context distinct from the actively managed environment of a compost pile designed for rapid biodegradation. Thus, this longer duration is essential for a comprehensive assessment of PBSA’s intrinsic hydrolytic stability.

### 2.2. Molecular Weight by Gel Permeation Chromatography (GPC)

**Figure 2:**
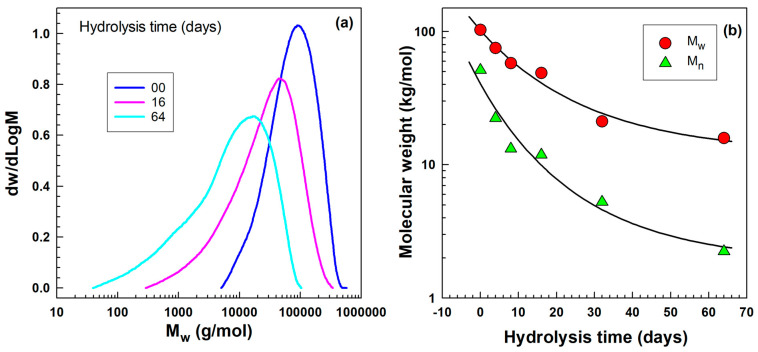
(**a**) Molecular weight distribution of PBSA after hydrolysis at 70 °C, (**b**) decrease in molecular weight during hydrolysis at 70 °C.

Before coming to detailed crystallization results it is beneficial to know the influence of hydrolysis at 70 °C on molecular weight distribution curves shown in Figure 2a for an original sample (00 days) and samples after 16 and 64 days of hydrolysis at 70 °C. The curves after hydrolysis are getting wider with increasing low molecular weight fraction. This fact is reflected in increased polydispersity index (PDI) being about 2, 4 and 7 for the original sample and 16 and 64 days samples, respectively.

In Figure 2b molecular weight (both M_n_ and M_w_ are shown) decreases gradually with the time of hydrolysis, initially the decrease is more pronounced.

The primary principle of degradation in our study is hydrolysis, which involves the cleavage of ester bonds [38] within the poly(butylene succinate-co-adipate) (PBSA) polymer backbone through reaction with water. Our analysis confirmed this ester cleavage by showing a clear decrease in molecular weight as the hydrolysis time increased, indicating the fragmentation of the polymer chains.

A similar decrease in molecular weight was observed, for example, by Tsuji and Okumura [39] who studied crystallization and hydrolytic/thermal degradation of a novel stereocomplexationable blend of poly(L-2-hydroxybutyrate) and poly(D-2-hydroxybutyrate).

### 2.3. DSC Analysis

#### 2.3.1. Initial Screening Experiments

Temperature 37 °C relates to temperatures used in usual biodegradation tests at anaerobic conditions; at 58 °C the biodegradation is usually tested at the composting conditions in aerobic environment but is also often tested at thermophilic anaerobic conditions. It is possible that during composting the temperature can rise even to higher temperatures, such as 70 °C.

**Figure 3:**
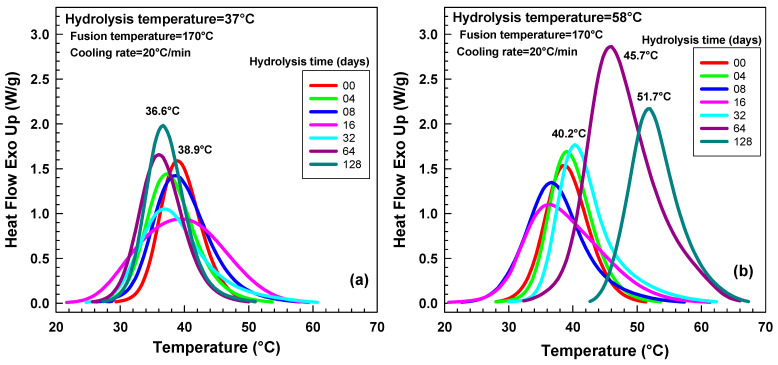
Normalized heat flow curves obtained by differential scanning calorimetry (DSC) during a 20 °C/min cooling rate for various hydrolysis times. (**a**) PBSA at 37 °C, (**b**) PBSA at 58 °C.

Figure 3a shows crystallization PBSA after hydrolysis at 37 °C. The crystallization peak position is in range 36 to 39 °C which is almost negligible. The hydrolysis temperature 58 °C (see Figure 3b) brought a little bit larger change in crystallization temperature changing from 40 to 45 and 51 °C for hydrolysis times 32, 64 and 128 days.

Our main objective was crystallization study after hydrolysis. In our initial screening experiment, we performed hydrolysis at 37, 58 and 70 °C. The weight loss values after 128 days at these temperatures were about 9, 54 and 72 wt. %, respectively. Corresponding shifts in crystallization temperature T_c_ were about 2, 12 and 25 °C, respectively. We found a very large change in crystallization after hydrolysis at 70 °C in a timeframe 0 to 64 days. Therefore, we decided to perform the hydrolysis again at 70 °C for a time period of 4, 8, 16, 32 and 64 days and to study the crystallization behavior in detail.

**Figure 4:**
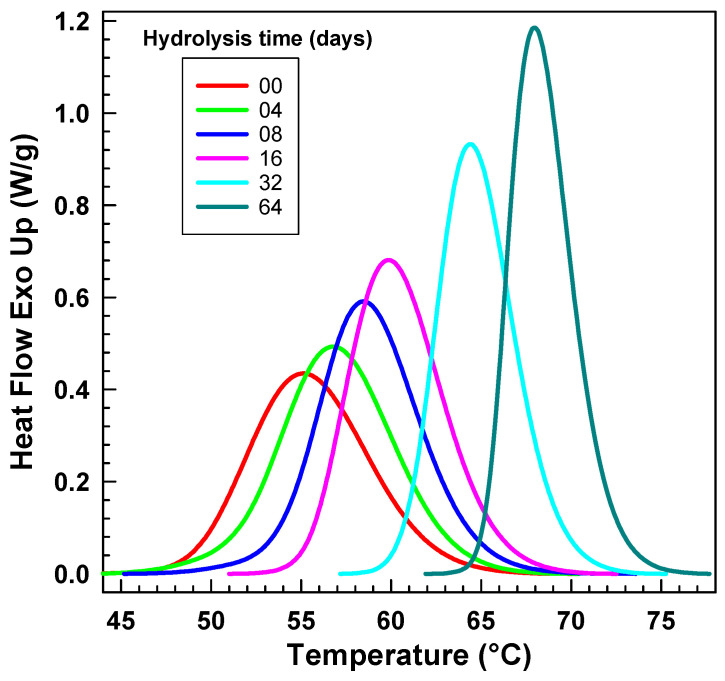
Normalized heat flow curves obtained by differential scanning calorimetry (DSC) during a 5 °C/min cooling ramp following hydrolysis at 70 °C.

Figure 4 illustrates a trend of increasing crystallization peak temperatures with progressing hydrolysis.

These findings align with observations reported by Rangari and Vasanthan [40], who investigated the crystallization behavior of poly(L-lactic acid) (PLLA) following enzymatic degradation. Their study documented an increase in crystallization temperature T_c_ from 89 °C to 96 °C and from 92 °C to 97 °C after 10 days of degradation for two separate PLLA samples. In our research, utilizing a cooling rate of 5 °C/min, we observed a similar increase in T_c_, specifically from 55 °C to 68 °C, over a 64-day hydrolysis period, representing a 13 °C increase. Furthermore, the peak height, normalized to sample size, exhibited a substantial increase from 0.44 W/g to 1.18 W/g, indicating a significant change in crystallization characteristics. The original heat flow data were recorded in milliwatts (mW). To normalize the results based on sample mass, the heat flow values were divided by the corresponding sample weight in milligrams (mg), yielding a final unit of watts per gram (W/g). This normalization procedure allows for a more direct comparison of thermal behavior across samples of varying mass.

Muthuraj et al. [41] reported an increase in T_c_ (from 81.1 to 96.5 °C) for poly(butylene adipate-co-terephthalate) (PBAT) but decreased T_c_ (from 92.0 to 77.1 °C) for poly(butylene succinate) (PBS) after 30 days conditioning at 50 °C with 90% RH. Our results are similar to PBAT (T_c_ increase) but are contrary to PBS (when T_c_ decreases).

Rabello and White [42] showed decreased T_c_ (from 115 to 107 °C) for polypropylene (PP) after 24 weeks of photodegradation which is the opposite of our increasing trend.

**Figure 5:**
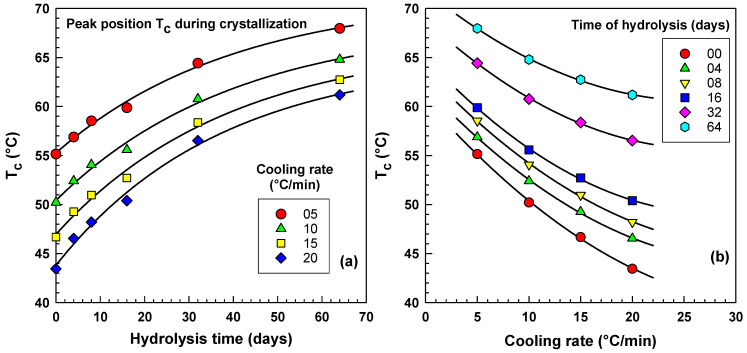
Crystallization peak temperature T_c_ as a function of (**a**) hydrolysis time at 70 °C and (**b**) cooling rate, as determined by differential scanning calorimetry (DSC).

An increase in crystallization temperature T_c_ was observed across all cooling rates tested (5, 10, 15, and 20 °C/min), as depicted in Figure 5a. Conversely, as shown in Figure 5b, higher cooling rates resulted in a shift of the crystallization temperatures towards lower values and the influence of cooling rate on T_c_ seems to be smaller after hydrolysis. This inverse relationship between cooling rate and crystallization temperature is consistent with observations reported in the literature. For example, Somrang et al. [43] documented a similar trend in their study of the crystallization kinetics of ethylene-acrylic acid copolymers. Likewise, Liu et al. [44] observed a decrease in crystallization temperature with increasing cooling rate during their investigation of the non-isothermal crystallization of biodegradable poly(butylene succinate-co-diethylene glycol succinate) copolymers. Le Delliou et al. [45] also observed T_c_ decrease with increasing cooling rate.

**Figure 6:**
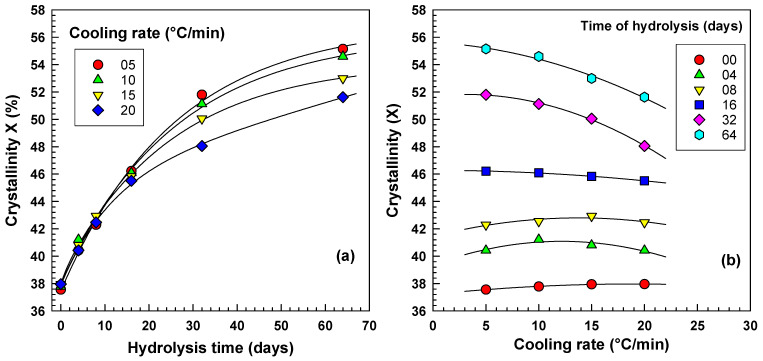
Crystallinity X from DSC cooling scan as a function of (**a**) hydrolysis time, (**b**) cooling rate.

The original heat flow curves illustrated in Figure 4 rendered not only the peak positions T_c_ but also the values of crystallinity when the theoretical enthalpy of melting for 100% crystalline PBS (110.3 J/g) [41] was used. The crystallinity increased significantly (from 38 to 54%) with increasing hydrolysis time (see Figure 6a).

The increase in crystallinity with increasing hydrolysis time shown in Figure 6a agrees well with Bikiaris et al. [37], who, in their investigation of the biodegradability of three poly(alkylene succinates), proposed that degradation predominantly occurs within the amorphous regions of the polymers, influencing their crystallinity. Their findings indicated that with prolonged hydrolysis, a marginal elevation in melting temperature was consistently observed, coupled with a notable increase in the associated heats of fusion. Specifically, after 60 days of hydrolysis, the heats of fusion for poly(ethylene succinate) (PESu) and poly(butylene succinate) (PBSu) were approximately 5 J/g greater than those of the respective undegraded polymers. Poly(propylene succinate) (PPSu), which exhibited the highest weight loss during biodegradation, showed a more substantial increase of 9 J/g in its heat of fusion. This increase is likely attributable to the preferential removal of amorphous material through hydrolysis, rather than a rise in crystallinity. Additionally, a slight shift of the polymer’s glass transition temperature towards higher values was noted. This observation aligns with the premise that the more mobile amorphous fractions are preferentially degraded, leaving behind a constrained phase located between crystallites.

Figure 6b reveals that crystallinity remained stable across all cooling rates during the initial 16 days of hydrolysis. This observation is in good agreement with Ren et al. [46] for PBSA. Subsequently, at 32 and 64 days, a clear inverse relationship emerged between cooling rate and crystallinity. Specifically, higher cooling rates resulted in lower crystallinity values. This suggests that during extended hydrolysis, slower cooling allows for enhanced molecular organization into lamellae. The 32 and 64 days results resemble that of Papadimitriou et al. [47] for poly(epsilon-caprolactone-co-propylene succinate) copolymers.

**Figure 7:**
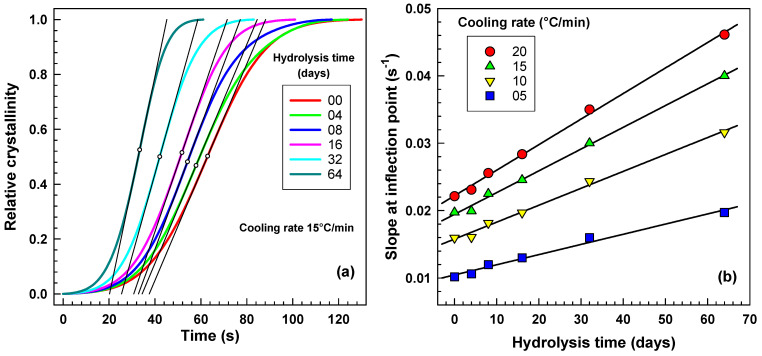
Crystallization kinetics from DSC. (**a**) relative crystallinity vs. time, (**b**) slope at the inflection point vs. time of hydrolysis.

The peaks of heat flow curves shown in Figure 4 get narrower with hydrolysis which suggests a change in crystallization kinetics. There are several possibilities how to get the kinetics of non-isothermal crystallization. Many researchers use modified Avrami equation that was originally formulated for isothermal crystallization. Other option is getting half time of crystallization when the relative crystallinity reaches 0.5 value. Kratochvil and Kelnar [48] suggest a slope of cumulative relative crystallinity curve in inflection point with temperature axis. In this case the slope does not have exactly the kinetical meaning. We have modified the Kratochvil method and obtained the slope at the inflection point in graph relative crystallinity versus time, see Figure 7a. This Figure 7a illustrates the evolution of relative crystallinity over time, represented as S-curves. As hydrolysis progressed, the overall crystallization time decreased possibly due to decreased amount of amorphous phase as it was shown in Figure 6. The crystallization kinetics are most accurately reflected by the slope at the inflection point of these curves. We have analyzed the data also by modified Avrami equation and half time of crystallization. All three analyses gave similar trends: increasing crystallization rate with increasing hydrolysis time for all cooling rates (5, 10, 15, 20 °C/min).

This slope, indicative of the crystallization rate, is plotted as a function of hydrolysis time in Figure 7b. Notably, the crystallization rate increased almost linearly with hydrolysis time across all measured cooling rates. Our results are in good agreement with other researchers.

The phenomenon of accelerated crystallization in shorter polymer chains was initially elucidated by Hoffman [49] in 1982, who proposed a mechanism involving reptation of molecules towards the growing lamellar front. Hoffman concluded that “the shorter molecules tend to enter the substrate at a more rapid rate than the longer ones”. Later in 1988 Hoffman and Miller [50] have demonstrated increasing crystallization rate with decreasing molecular weight on series of polyethylene samples and wrote that “the chain being drawn onto the crystal by the force of crystallization exerts a resistance to this force that is essentially proportional to its length”.

Since then, numerous studies have corroborated this observation, demonstrating an increase in crystallization rate with decreasing molecular weight for a variety of polymers, including polyethylene [50], polypropylene [51], polycaprolactone [52], polycarbonate [53], poly(butylene adipate) [54], poly(trimethylene terephalate) [55], poly(L-lactide) [56], poly(ethylene succinate) [57], and poly(ethylene adipate) [58].

#### 2.3.2. The Avrami Analysis of Non-Isothermal Crystallization

In 1940, Avrami [59] provided a description of isothermal crystallization:

(1) $$V=1-e^{-Bt^{k}}$$

where $V=V\left(t\right)$ represents the volume of the new phase per unit volume of space, B is a function of temperature and k is a constant which can have value 1, 2, 3 or 4 reflects the growth morphology, including linear, plate-like, and polyhedral development. Isothermal crystallization typically exhibits a sigmoidal (S-shaped) curve, characterized by an initial slow phase, followed by rapid growth, and eventually reaching a plateau.

Jeziorny later adapted the classical Avrami equation to accommodate non-isothermal crystallization processes:

(2) $$X\left(t\right)=1-\exp\left(-Z_{t}\cdot t^{n}\right)$$

(3) $$1-X\left(t\right)=\exp\left(-Z_{t}\cdot t^{n}\right)\;$$

(4) $$ln\left[1-X(t)\right]=-Z_{t}\cdot t^{n}\;\;\;\;\;\;\;\;\;$$

(5) $$-ln\left[1-X(t)\right]=Z_{t}\cdot t^{n}$$

(6) $$log\left\{-ln\left[1-X(t)\right]\right\}=\log Z_{t}+n\cdot \log t\;$$

where n is the Avrami exponent which is linked to both the nucleation mechanism and the geometry of crystal growth and $Z_{t}$ is the kinetic growth rate constant in the non-isothermal crystallization process. To account for the impact of varying cooling rates on non-isothermal crystallization process, proposed a correction for the rate parameter: $Z_{t}$ should be corrected as follows:

(7) $$\log Z_{c}=\frac{\log Z_{t}}{\phi }\;$$

where $Z_{c}$ is the modified crystallization rate constant with respect to the cooling rate ϕ [60].

Avrami equation was originally developed for the study of isothermal crystallization of low molecular substances. The n index is an integer ranging from 1 to 4 and is related to nucleation and growth parameters as follows:

(8) $$n=\alpha +\frac{d}{p}$$

Originally formulated for studying isothermal crystallization in low molecular weight substances, the Avrami equation uses an integer index n (ranging from 1 to 4) that is related to nucleation and growth characteristics through the Equation (8). Here, α denotes the nucleation index (1 for homogeneous and 0 for heterogeneous nucleation), d represents the dimensionality of crystal growth (1, 2, or 3 for one-, two-, and three-dimensional growth, respectively), and p is the growth index (1 for interface-controlled and 0.5 for diffusion-controlled growth). Although initially designed for isothermal conditions, the Avrami equation has been widely applied by researchers to analyze polymer crystallization kinetics under non-isothermal conditions. In such cases, the Avrami parameters lose their direct physical interpretation due to the influence of temperature changes on both nucleation and growth rates. Instead, they serve as adjustable parameters for fitting the Avrami equation to experimental data. Nonetheless, these parameters remain valuable for monitoring alterations in polymer crystallization behavior resulting from modifications like hydrolysis [61].

**Figure 8:**
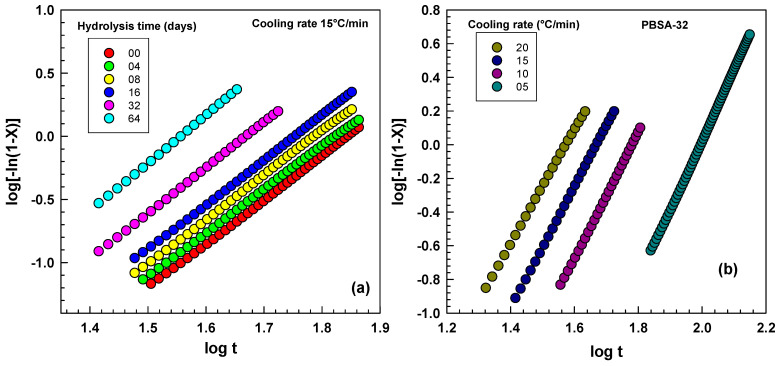
Avrami plot from DSC. (**a**) for various hydrolysis times at cooling rate 15 °C/min, (**b**) PBSA after 32 days of hydrolysis at 70 °C for various cooling rates (5–20 °C/min).

Figure 8a depicts Avrami plot for various hydrolysis times. The lines for 0–16 days are close together but the lines for 32 and 64 days are quite separated witch signifies a large change in crystallization kinetics. From the intercept one can derive Jeziorny’s parameter $Z_{t}$. Figure 8b shows four lines for PBSA after 32 days of hydrolysis for various cooling rates. While the lines for cooling rates 20,15 and 10 °C/min are close together the line for 5 °C/min is in greater distance from these three which reflects large change in crystallization kinetics. Our results show similar trends to Jiang et al. who studied PBS/PBSA blends [62].

**Figure 9:**
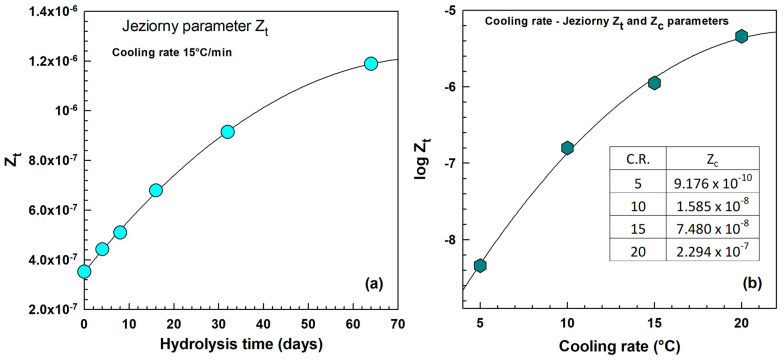
Plot of Jeziorny’s parameter $Z_{t}$ from Avrami plot. (**a**) as a function of hydrolysis times at cooling rate 15 °C/min, (**b**) PBSA after 32 days of hydrolysis at 70 °C as a function of cooling rate.

Figure 9a shows a crystallization kinetics parameter $Z_{t}$ as a function of hydrolysis time. The growth rate steadily increases with hydrolysis time initially up to 16 days it seems to be a linear relationship. Figure 9b illustrate the kinetic parameter $Z_{t}$ as a function of cooling rate with increasing trend. Our results agree well with Bandyopadhyay and Ray who observed increase in $Z_{t}$ with increasing cooling rate for polypropylene/PBSA blends [63] and Figure 9b also contains the corrected parameter $Z_{c}$ calculated according to Equation (7) which increases as well. Chen et al. also observed similar trend in $Z_{c}$ for biodegradable polyester poly(3-hydroxybutyrate) (PHB) [64].

Numerous researchers [65,66,67,68,69,70] have employed Ozawa analysis to investigate non-isothermal crystallization kinetics. This method necessitates the use of closely spaced cooling rates to achieve a linear relationship. The Ozawa equation is written as

(9) $$X=1-e^{\left[\frac{-K\left(T\right)}{\phi^{m}}\right]}$$

where X is relative crystallinity in range 0–1, m is the Ozawa parameter illustrating the nucleation and growth, $K\left(T\right)$ is Ozawa cooling function representing rate of crystallization and ϕ is the cooling rate. For the quantitative analysis it is convenient to use a double logarithmic form of this equation as follows:

(10) $$\log\left[-ln\left(1-X\right)\right]=\log K\left(T\right)-m\log\phi$$

In the study of non-isothermal crystallization kinetics, particularly within the Ozawa model, the Ozawa exponent (m) serves a role analogous to the Avrami exponent (n) in isothermal analysis, offering insights into the spatial characteristics of crystal development. Theoretically, for idealized growth patterns, the value of m is expected to be an integer directly corresponding to the dimensionality of crystal propagation: a value of 1 suggests one-dimensional growth, such as the formation of needle-like or rod-shaped crystals; a value of 2 indicates two-dimensional growth, exemplified by plate-like or disc-shaped morphologies; and a value of 3 is associated with three-dimensional growth, commonly observed in spherulitic structures.

**Figure 10:**
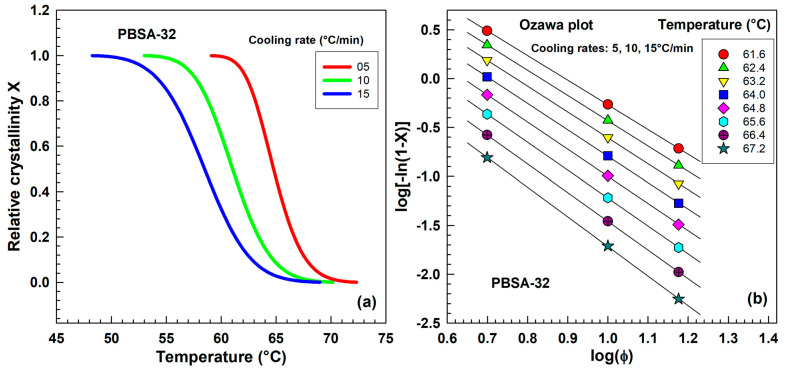
DSC results: (**a**) Relative crystallinity as a function of temperature at three cooling rates for PBSA-32 (32 days of hydrolysis at 70 °C), (**b**) Ozawa plot.

**Figure 11:**
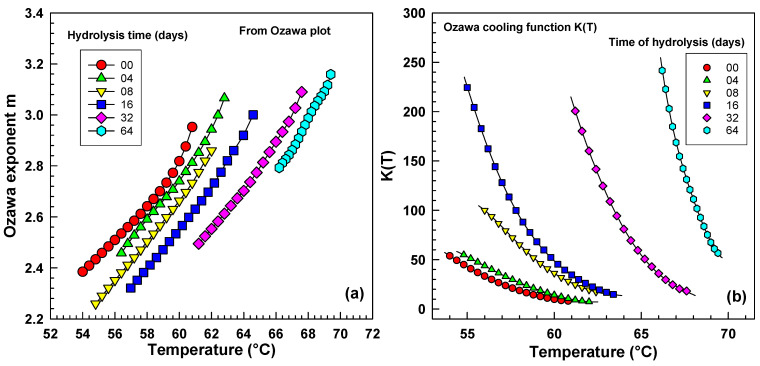
Detailed analysis of Ozawa parameters obtained from Figure 10b: (**a**) Ozawa exponent m for PBSA, (**b**) Ozawa cooling function.

Figure 10a illustrates relative crystallinity X as a function of temperature at three cooling rates for PBSA-32 (32 days of hydrolysis at 70 °C). For every temperature three values of relative crystallinity are read and these values are presented in Figure 10b in logarithmic coordinates. Three rates were chosen according to Ozawa experiment. Somsunan and Mainoiy [71] in their study of non-isothermal crystallization of polylactid acid/PBS blends used seven cooling rates in the Ozawa plot and the relationship was not linear. Ozawa himself [72] utilized cooling rates of 1, 2, and 4 K/min. Figure 10b presents the Ozawa plot derived from three cooling rates, demonstrating excellent linearity. The data were plotted according to Equation (10). Intercept gives value $\log K\left(T\right)$ and slope gives the value m. These parameters were plotted as a function of temperature in Figure 11a,b.

Figure 11a shows is the Ozawa parameter m illustrating the nucleation and growth. It has a value from 2.2 till 3.2 and shifts towards higher temperatures with progressing time of hydrolysis. Ozawa parameter m has different value for different temperatures. At higher temperature the Ozawa parameter m is higher. Also, the range seems to increase, e.g., for 64 days of hydrolysis it is 2.8–3.2 compared to 0 days of hydrolysis when the range is 2.4–3.0.

Figure 11b displays the Ozawa cooling function, K(T), for samples subjected to varying degrees of hydrolysis. The Ozawa cooling function serves a dual purpose: it delineates the temperature range within which crystallization occurs and quantifies the crystallization kinetics. Following hydrolysis, a shift in crystallization temperatures towards higher values was observed. Additionally, the K(T) values were elevated for samples hydrolyzed for 16, 32, and 64 days compared to those hydrolyzed for 0, 4, and 8 days.

### 2.4. Polarized Optical Microscopy Results

**Figure 12:**
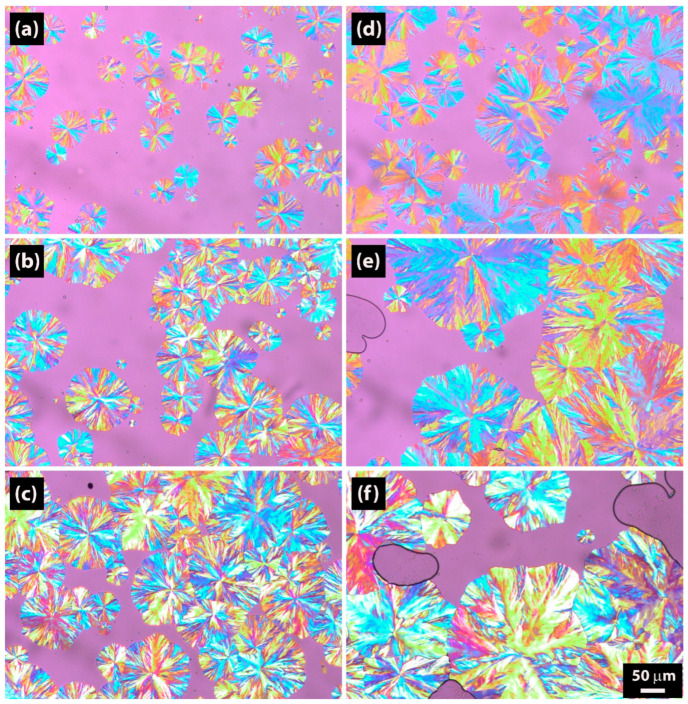
Non-isothermal crystallization by polarized optical microscopy at 2 °C/min cooling rate: (**a**) PBSA-00 at 59.7 °C, (**b**) PBSA-04 at 60.6 °C, (**c**) PBSA-08 at 59.4 °C, (**d**) PBSA-16 at 63.2 °C, (**e**) PBSA-32 at 63.8 °C, (**f**) PBSA-64 at 68.7 °C. The pink color represents amorphous phase, the spherulites are the blue and yellow circles and the black line is a boarder of cavity (vacuum) created during the cooling.

Figure 12 illustrates the morphological changes in spherulitic structures resulting from hydrolysis. Samples subjected to shorter hydrolysis periods retained their spherical morphology, whereas those subjected to prolonged hydrolysis exhibited dendritic structures, a phenomenon consistent with observations reported by Nie et al. [73] for random poly(p-dioxanone-co-butylene-co-succinate) copolyesters. The spherulitic morphologies observed in our PBSA samples can be compared with those reported in studies on the crystallization of other copolyesters, such as those by Qiu et al. [74], Xue and Qiu [75], Liu et al. [76], Yang and Qiu [77], and Papageorgiou and Bikiaris [78].

**Figure 13:**
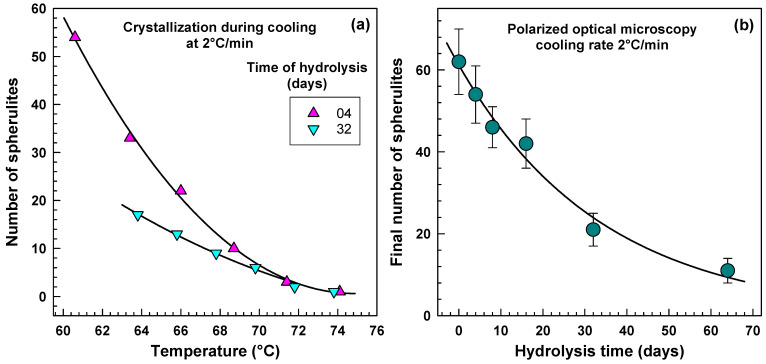
Number of spherulites from polarized optical microscopy: (**a**) comparison of the number of spherulites for PBSA-04 and PBSA-32 during non-isothermal crystallization at 2 °C cooling rate, (**b**) final number of spherulites vs. hydrolysis time.

Figure 13a demonstrates a decline in the number of spherulites with increasing temperature for both 4 and 32 days of hydrolysis. Reduced spherulite number density at higher hydrolysis temperatures, aligning with nucleation theory reported by Crist and Schultz [79] who stated that the primary nucleation density, which corresponds to the number of spherulites, decreases at higher crystallization temperatures, signifies a coarser microstructure. Larger spherulites typically correlate with increased brittleness, decreased mechanical strength, and potentially higher opacity due to altered light scattering. This morphological shift suggests a trend toward a more mechanically fragile and optically less transparent material post-hydrolysis.

Jariyavidyanont et al. [80] conducted a study on homogeneous crystal nucleation in poly(butylene succinate-ran-butylene adipate). Their research indicated that the studied PBSA copolymer exhibited a crystallization rate approximately ten times slower than the PBS homopolymer. It is conceivable that during the hydrolysis the copolymer composition changes, the amount of adipic units decrease and the final copolymer is more similar to PBS. In this way one could explain faster crystallization of PBSA after hydrolysis.

Figure 13b illustrates a decrease in the number of spherulites as hydrolysis progresses. It is plausible that the hydrolysis process disrupts nucleation centers, resulting in a reduced number of larger spherulites.

Figure 13b presents data derived from numerous cooling experiments, with examples illustrated in Figure 12. To determine the average values and standard deviations for the final number of spherulites, three cooling experiments were consistently performed for each condition. It is important to note that the standard deviations tend to be higher for shorter hydrolysis times, where a larger number of spherulites were observed. Conversely, the standard deviations are considerably smaller for hydrolysis times of 32 and 64 days, conditions under which the number of spherulites was significantly lower.

**Figure 14:**
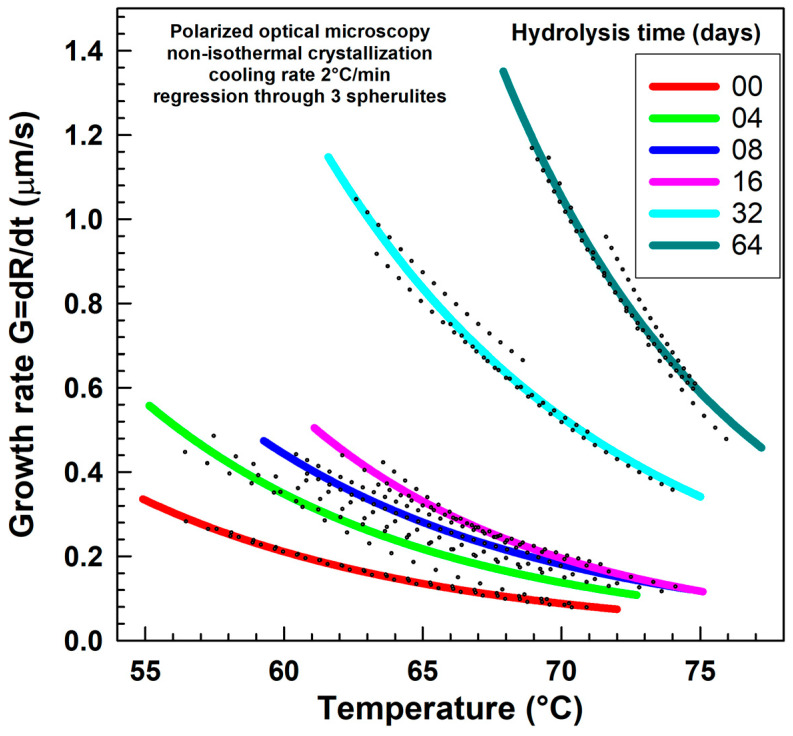
Spherulite growth rate as a function of temperature during non-isothermal crystallization at a cooling rate of 2 °C/min.

Figure 14 illustrates the effect of hydrolysis time on crystallization kinetics, as determined by non-isothermal polarized optical microscopy. Traditionally, the spherulite growth rate G is determined under isothermal conditions by analyzing the slope of spherulite radius plotted against crystallization time. However, Chen and Chung [81] proposed an alternative approach for non-isothermal solidification, suggesting that G can be approximated by calculating the first derivative of the spherulite radius versus temperature graph at each data point. This methodology has been employed by several other researchers [82,83,84,85].

During the cooling phase of the experiment, the temperature was decreased at a constant rate of 2 °C/min. Throughout the experiment, the temperature was continuously monitored using a web camera directed at the LINKAM temperature controller, ensuring that each was associated with a precise temperature reading. Images were acquired at 5-s intervals for subsequent analysis. For each hydrolysis time point, three individual spherulites were selected, and their radius was measured as a function of time. The derivation of this curve is the growth rate G=dR/dt with units of μm/s_._ The black points in Figure 14 represent the derivation G for each individual spherulite and are a little bit scattered. Throughout the scattered black points, the colored regression line was drawn.

We observed a strong correlation between our DSC in Figure 11b and POM experiments (see Figure 14), specifically when comparing the Ozawa cooling function, K(T), and the non-isothermal crystallization kinetics. The trends displayed in these graphs, derived from two independent measurement techniques, exhibited a remarkable similarity. While POM provides a visual representation of the planar growth of a limited number of spherulites, DSC measures the bulk crystallization of the entire spherulite population. We wish to emphasize this significant finding, as, to our best knowledge, such a direct connection between these two methods has not been previously demonstrated.

**Figure 15:**
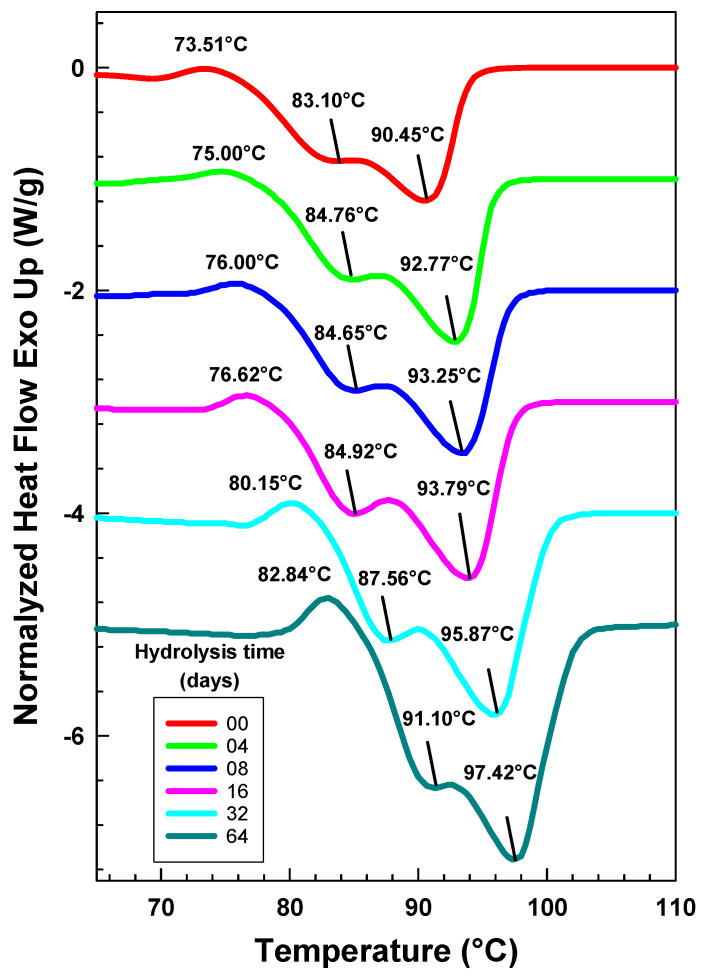
Change in two melting points T_m_ and cold crystallization temperatures by DSC at heating rate 20 °C/min.

Figure 15 illustrates the systematic variation in melting point T_m_ during hydrolysis. There are three peaks present. The right peak is the major peak, but the left peak is also important. The presence of two melting peaks in copolymer indicates presence of two lamellar thicknesses. The precise values are shown in Figure 15. It is worth mentioning the existence of exothermal cold-crystallization peak. Its values were also recorded.

A notable shift from approximately 90 °C to 97 °C was observed. Lindstrom et al. [86] developed a solid-phase extraction method for the simultaneous extraction of adipic acid, succinic acid, and 1,4-butanediol, which are products of poly(butylene adipate) and poly(butylene succinate) hydrolysis. Their findings indicated a higher concentration of adipic acid compared to succinic acid under identical hydrolysis conditions. The initial characterization of PBSA copolymers was reported by Ahn et al. [87] in 2001, who synthesized and analyzed copolymers of succinic acid, adipic acid, and 1,4-butanediol. They reported a melting point T_m_ of 114 °C for pure poly(butylene succinate) and 92 °C for PBSA with 21% adipic acid (determined by NMR). Perez-Camargo et al. [88] observed a similar trend. Baidurah et al. [30,89] demonstrated a change in copolymer composition following thermally assisted PBSA hydrolysis, specifically a reduction in adipic acid content. Our observation of increased T_m_ after hydrolysis suggests a corresponding alteration in copolymer composition. Specifically, hydrolysis leads to a decrease in molecular weight and a shift towards a lower adipic acid content. Marten et al. [38] in their studies on the enzymatic hydrolysis of polyesters reported significant hydrolysis in polyester containing adipic units while the one with succinic units did not hydrolyze at all at tested temperatures 25, 37 and 50 °C.

**Figure 16:**
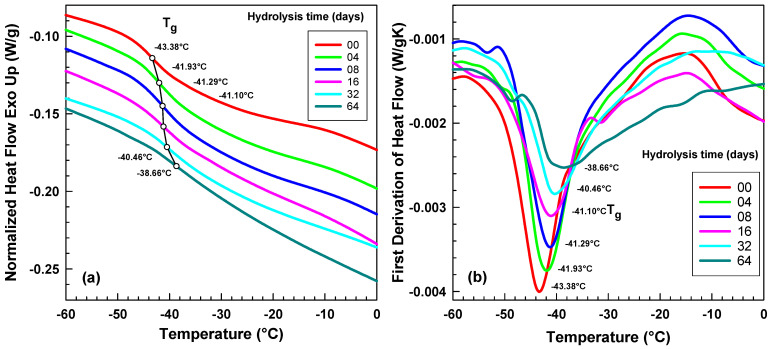
Change in glass transition temperature T_g_ by DSC at heating rate 10 °C/min: (**a**) original heat flow curves, (**b**) first derivation of heat flow curves.

Figure 16 illustrates the variation in glass transition temperature T_g_ as hydrolysis progresses. Figure 16a presents heat flow curves, normalized to sample size and slightly offset for improved clarity, while Figure 16b displays the first derivative curves of heat flow, where the peak position corresponds to T_g_, allowing for straightforward determination. Ahn et al. [87] reported T_g_ values for PBSA copolymers with compositions 79/21, 90/10, and 100/0 (succinic acid/adipic acid), which were −44 °C, −37 °C, and −33 °C, respectively. Perez-Camargo et al. [88] reported T_g_ values of −43.7 °C and −35.2 °C for PBSA copolymers with compositions 80/20 and 100/0 (succinic acid/adipic acid), respectively. In our study, T_g_ values shifted from −43.38 °C to −38.66 °C during hydrolysis.

The change of glass transition temperature T_g_ shown in Figure 16 with progressing hydrolysis evokes the idea of the change in copolymer composition which is consistent with the explanation provided for Figure 15. Change in PBSA copolymer composition during soil biodegradation has been reported by Baidurah et al. (2012) [30]. Originally the composition obtained by thermally assisted hydrolysis and methylation-gas chromatography (THM-GC) was 82.2/17.8 (butylene succinate/butylene adipate BS/BA). After 28 days of soil biodegradation test the ratio was 85.5/14.5 (BS/BA), so the content of butylene adipate decreased from 17.8 to 14.5 mol %. Or in another words the content of butylene succinate (BS) increased from 82.2 to 85.5 mol %. Baidurah et al. (2013) [89] performed a comparison of biodegradation of original film compared to heated film. By DSC they have obtained heat of fusion 53.0 and 48.6 J/g for the original and heated film samples, respectively. Then, the degree of crystallinity of PBSA was calculated from the measured value of the heat of fusion divided by the theoretical value for the 100% crystalline copolyester according to the method reported by Tserki et al. [90,91] who used the theoretical heat of fusion values for PBSu and PBAd 110.5 and 135.0 J/g, respectively. The obtained degree of crystallinity for the original and heated film samples were 46.1 and 42.4%, respectively, indicating that crystallinity of the PBSA film was considerably lowered by heat and cool treatment. Then the weight loss for the original film was 31.9 wt. % while for the less crystalline heated film it was 85.6 wt. %.

Now we have observed during hydrolysis a gradual change in glass transition temperature T_g_ from −43.38 up to −38.66 °C. Based on the data reported by Ahn and al. [87] and Tserki et al. [90,91] we can assume a shift in copolymer composition towards higher butylene succinate BS content, or lower butylene adipate BA content. A rough estimation suggests that the composition of our samples shifted from approximately 80/20 to 90/10 (succinic acid/adipic acid) during the hydrolysis.

## 3. Materials and Methods

### 3.1. Materials

Poly(butylene succinate-co-adipate) (PBSA), with the product name Bionolle 3001 MD, was used in this study. This material was supplied by Showa Denko, Tokyo, Japan, and had a melt flow index (MFI) of 3 g/10 min and an original molecular weight M_w_ = 1.08 × 10^5^ g/mol. Product literature lists tensile stress at break (ISO 527-3 [92]) 40 MPa, elongation at break 780%, Young’s modulus 320 MPa, density 1.23 g/cm^3^, heat of combustion 23.9 kJ/g, heat distortion temperature (HDT) at 0.45 MPa is 69 °C, degree of crystallinity is in range 20–35%, melting point 94 °C and glass transition temperature is −45 °C.

All chemicals for hydrolysis in phosphate buffered saline testing came from Merck KGaA, Darmstadt, Germany.

**Scheme 1:**
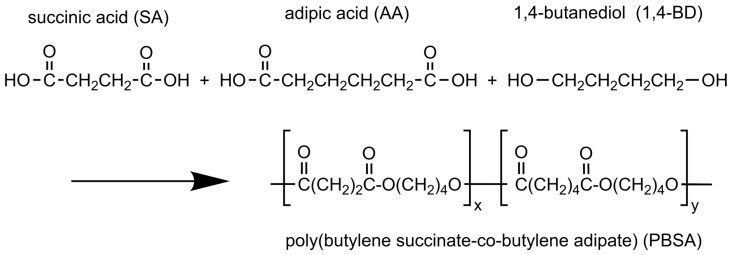
Chemical structure of succinic acid, adipic acid, 1,4-butanediol and poly(butylene succinate-co-butylene adipate) copolymer (PBSA).

Scheme 1 shows the chemical structure of poly(butylene succinate-co-adipate) and its constituents.

### 3.2. Compression Molding

Following ASTM D4703 [93], compression molding was performed to produce test sheets of 0.2 mm thickness. A stainless-steel frame, measuring 4 cm × 4 cm, was used to contain the material. Polymer pellets were preheated at 120 °C, preheating time was 3 min under minimal contact pressure. Subsequently, the material was compression molded at 10 MPa, molding time was 2 min using a hydraulic press, molding temperature was 120 °C. The resulting sheets were then quenched under pressure using a separate cold press. Average cooling rate was 25 °C/min.

### 3.3. Hydrolysis in Phosphate Buffered Saline

The phosphate buffered saline was prepared this way: we prepared 800 mL of distilled water in a suitable container, we added 8 g of NaCl to the solution, 200 mg of KCl was added to the solution, 1.44 g of Na_2_HPO_4_ was added to the solution, 240 mg of KH_2_PO_4_ was added to the solution, pH was adjusted to 7.2, and finally we added distilled water until volume was 1 L.

#### Hydrolysis at Elevated Temperature

Polymer films were cut into 5 × 5 mm pieces, and then 200 mg of polymer was weighed into a 250 mL biometric flask. A 200 mL portion of phosphate buffer (pH 7.2) was added to each flask. To prevent biodegradation, a microbial growth inhibiting substance (NaN_3_) was included in the buffer solution. The long-term hydrolysis experiment was conducted in a Memmert UFE 400 oven (Memmert, Schwabach, Germany) equipped with a digital controller maintaining a temperature variation of ± 1 °C. The experimental temperatures were set at 37 °C, 58 °C, and 70 °C. The contents of the flasks were stirred continuously throughout the one-day test period.

At regular time intervals of 4, 8, 16, 32, and 64 days, water samples were collected from the flasks to quantify the amount of polymer that transitioned from the solid phase into the liquid phase due to hydrolysis. This was determined by measuring the organic carbon released from the solid PBSA polymer phase into the soluble phase.

Volumes of approximately 50 µL of each collected liquid sample, maintained at room temperature, were introduced in triplicate into a Shimadzu TOC 5000A Analyzer (Shimadzu, Kyoto, Japan) for dissolved organic carbon analysis. The results were expressed in mg/L.

The degree of hydrolysis was also defined as the mass loss of the solid sample after its removal from the buffer solution and subsequent weighing.

(11) $$H=\frac{c_{DOC}.V}{m\cdot w_{c}}$$

where

H is degree of hydrolysis PBSA [%];
$c_{DOC}$ total organic carbon in liquid after hydrolysis [mg/L];
m the weight of the PBSA sample used in the hydrolysis [mg];
$w_{c}$ theoretical carbon content in the tested film PBSA film [%];
V the total volume of the liquid phase in the bottle [L].

### 3.4. Gel Permeation Chromatography (GPC)

Gel permeation chromatography (GPC) was used to obtain the weight average molecular weight (Mw) and molecular weight distribution before and after hydrolysis. HT-GPC 220 system (Agilent Technologies, Santa Clara, CA, USA) with dual detection system (“VIS” viscosity detectors and “RI” refractive index) was used. The refractive index (RI) detector quantifies the change in refractive index of the eluent, directly correlating with the macromolecular concentration. The viscosity (VIS) detector measures the intrinsic viscosity, a parameter sensitive to the hydrodynamic volume and conformation of the polymer chains in solution. The synergistic application of RI and VIS detection enables the determination of absolute molecular weight and provides insights into macromolecular architecture, surpassing the limitations of concentration-dependent detectors alone. This dual detection methodology facilitates a more rigorous and comprehensive characterization of the polymeric species. THF was used as a solvent, concentration was about 2 mg/mL. Detection and separation were performed in mixed columns from Polymer Laboratories, 300 × 7.8 mm. Solvent was THF, temperature 40 °C, loading volume 100 μL, flow rate 1.0 mL/min. Calibration was performed with narrow polystyrene standards (Polymer Laboratories Ltd., Church Stretton, Shropshire, UK) having molecular weight in range 580 to 3,000,000 g/mol.

### 3.5. Differential Scanning Calorimetry (DSC)

A Differential scanning calorimeter DSC1 by Mettler Toledo, Columbus, OH, USA was used to identify characteristic peaks. About 7 mg of the sample was inserted into the aluminum crucible and heat flow was measured in a N_2_ (flow rate 200 mL/min). For $T_{g}$ evaluation experiment the temperature increased from −90 °C to +25 °C. For non-isothermal crystallization experiments the experiment started at 25 °C with heating ramp 20 °C/min to 140 °C, the next step was cooling from 140 °C to −20 °C at cooling rates 20, 15, 10, and 5 °C/min. Three measurements were always averaged and the standard deviation was less than 2%.

### 3.6. Polarized Optical Microscopy (POM)

A Polarized optical microscope Olympus BX41 (Olympus, Tokyo, Japan) together with a Linkam TP 94 hot stage (Linkam Scientific Instruments, Salfords, UK) were used for spherulitic crystallization kinetics evaluation at a constant cooling rate.

## 4. Conclusions

Our preliminary study of hydrolysis at 37, 58, and 70 °C showed a dramatic increase in degradation rate with temperature. Unlike other research at lower temperatures, this study comprehensively analyzes PBSA hydrolysis at 70 °C in phosphate-buffered saline, revealing significant alterations in structural, thermal, and morphological characteristics. GPC showed that hydrolysis reduced weight-average molecular weight (Mw) and substantially increased the polydispersity index (PDI) from ~2 to 7 after 64 days. DSC showed that hydrolysis increased the crystallization peak temperature $\left(T_{c}\right)$ from 55 °C to 68 °C and crystallinity from 38% to 54%. The increase in crystallinity was most likely caused by hydrolysis of amorphous regions. Cold crystallization temperature changed from 73.51 °C to 82.84 °C, melting temperature $\left(T_{m1}\right)$ shifted from 83.10 °C to 91.10 °C, melting temperature $\left(T_{m2}\right)$ shifted from 90.45 °C to 97.42 °C, and the glass transition temperature $\left(T_{g}\right)$ shifted from −43.38 °C to −38.66 °C, suggesting altered copolymer composition due to greater susceptibility of adipic acid units to hydrolysis than succinic acid units. Non-isothermal crystallization kinetics, evaluated by analyzing the slope of the cumulative relative crystallinity curve at its inflection point versus time, effectively captured accelerated crystallization with increasing hydrolysis time. This new method, consistent with traditional analyses (Jeziorny modified Avrami equation), showed an increase in crystallization rate with hydrolysis time, providing a more direct quantification of crystallization kinetics. POM showed a morphological transition from well-defined spherulites in early hydrolysis to less organized, dendritic structures with prolonged exposure. Lower nucleation rates resulted in fewer, larger spherulites.

## Data Availability

The original contributions presented in this study are included in the article. Further inquiries can be directed to the corresponding author(s).
